# NUTRITION THERAPY IN DISCHARGED OLDER ADULTS REDUCES HOSPITAL READMISSIONS AND LENGTH OF STAY

**DOI:** 10.1093/geroni/igad104.2104

**Published:** 2023-12-21

**Authors:** Alfons Ramel

**Affiliations:** University of Iceland, Reykjavik, Hofuoborgarsvaoio, Iceland

## Abstract

The aim was to investigate effects of six-month nutrition therapy on hospital readmissions, LOS, mortality and need for long-term care residence up to 18-months post-discharge in older Icelandic adults. Participants (>65 years) were randomised into intervention (n=53) and control (n=53) before discharge from a geriatric unit. The intervention group received nutrition therapy based on the Nutrition Care Process, including home visits, phone calls, freely delivered energy- and protein-rich foods and supplements for six months after hospital discharge. The Icelandic electronic hospital registry was accessed to gain information on emergency room visits (ER), hospital readmissions, LOS, mortality and need for long-term care residence. One subject from each arm dropped out during the intervention period. The intervention increased body weight (+5.2kg, 95%CI: 3.9-6.5kg) and energy intake (1696kcal, 95%CI: 1557-1834kcal) compared to control. The intervention group had a lower proportion of participants with at least one readmission compared to control (1 month: 1.9% vs 15.8%, P=0.033; 6 months: 25.0% vs 46.2%, P=0.021; 12 months: 38.5% vs 55.8%, P=0.051; and 18 months: 51.9% vs 65.4%, P=0.107). There was also a lower total number of readmissions (significant at 1, 6 and 12 months) and a shorter LOS (significant at all time points) in the intervention group. However, there were no differences between groups in ER visits, mortality and need for long-term care residence. It is of great clinical relevance that a six-month nutrition therapy in older Icelandic adults discharged from hospital reduced hospital readmissions and shortens LOS at the hospital up to 18-months post-discharge.

